# Secreted and Transmembrane αKlotho Isoforms Have Different Spatio-Temporal Profiles in the Brain during Aging and Alzheimer's Disease Progression

**DOI:** 10.1371/journal.pone.0143623

**Published:** 2015-11-24

**Authors:** Anna Massó, Angela Sánchez, Lydia Gimenez-Llort, Jose Miguel Lizcano, Manuel Cañete, Belen García, Virginia Torres-Lista, Meritxell Puig, Assumpció Bosch, Miguel Chillon

**Affiliations:** 1 Departament Bioquímica i Biologia Molecular, Universitat Autònoma Barcelona, Bellaterra, Spain; 2 Center of Animal Biotechnology and Gene Therapy (CBATEG), Universitat Autònoma Barcelona, Bellaterra, Spain; 3 Institut de Neurociencies, Universitat Autònoma Barcelona, Bellaterra, Spain; 4 Departament de Psiquiatria i Medicina Legal, Universitat Autònoma de Barcelona, Bellaterra, Spain; 5 Institució Catalana de Recerca i Estudis Avançats (ICREA), Barcelona, Spain; Inserm U837, FRANCE

## Abstract

The Klotho protein is a β-glucuronidase, and its overexpression is associated with life extension. Its mechanism of action is not fully understood, although it has been recently reported that αKlotho improves synaptic and cognitive functions, and it may also influence a variety of structures and functions during CNS maturation and aging. The αKlotho gene has two transcripts, one encoding a transmembrane isoform (m-KL), and the other a putative secreted isoform (s-KL). Unfortunately, little is known about the secreted αKlotho isoform, since available antibodies cannot discriminate s-KL from the KL1 domain cleaved from the transmembrane isoform. This study shows, for the first time, that the *klotho* transcript produced by alternative splicing generates a stable protein (70 kDa), and that in contrast to the transmembrane Klotho isoform, it is ten times more abundant in the brain than in the kidney suggesting that the two isoforms may have different functions. We also studied whether *klotho* expression in the CNS was influenced by aging, Alzheimer's disease (AD), or a healthy lifestyle, such as voluntary moderate continuous exercise. We observed a strong correlation between high expression levels of the two *klotho* transcripts and the healthy status of the animals. Expression of Klotho in brain areas decayed more rapidly in the 3xTg-AD model of AD than in healthy animals, whilst moderate continuous exercise in adulthood prevents the decline in expression of both *klotho* transcripts.

## Introduction

Several studies in mice have revealed that the mutation of the single gene *klotho* on chromosome 13 induces a process of accelerated aging [1, 2], and mice died prematurely around 2 months of age [1]. In contrast, transgenic mice overexpressing this gene had between 30% and 40% greater life expectancy [3]. In mice and humans, the *klotho* gene encodes a transcript of 5.2 kb [1]. In the third exon, there is an alternative splicing donor site that can generate two different transcripts: one encoding a transmembrane form (full-length transcript, 1014 amino acids), and the other, a secreted form of the protein (truncated transcript, 550 amino acids). The full-length transcript encodes a single pass transmembrane protein with a molecular weight of approximately 130 kDa (m-KL). The transcript generated by alternative splicing generates a secreted form of the protein (s-KL) that is formed solely by the KL1 domain, with an approximate weight of 70 kDa. However, there is some controversy regarding the s-KL protein because, to date, it has not been detected in body fluids using antibodies [4].

In the brain, *klotho* is highly expressed in the ependymal cells of the choroid plexus and in Purkinje EC cells, as well as in hippocampal neurons [1], although Clinton et al. have recently demonstrated that *klotho* mRNA and protein are detected throughout the brain parenchyma, co-localizing in neurons and oligodendrocytes during early postnatal development [5]. This suggests that Klotho protein may influence a variety of structures and functions during CNS maturation and aging [6]. Compared to wild-type mice, kl-/kl- mice have fewer dopaminergic neurons [7], neuronal degeneration in the hippocampus [8], hypomyelination [9], fewer synapsys and lower synapse-related protein levels [8, 10], and deficient axonal transport [11]. In addition, behavioral studies indicate that kl-/kl- mice also have deficits in memory retention [12], probably due to an increase in oxidative stress in the brain. In contrast, mice overexpressing Klotho performed better in the Morris Water-Maze test, which evaluates hippocampus-dependent spatial memory and learning. Of note, Dubal et al. reported that Klotho likely has beneficial effects on cognitive functions, independently of ageing, through a mechanism involving the synaptic NMDA receptor (NMDAR) subunit GluN2B [6].

In addition, recent studies conducted in three independent human cohorts showed that carriers of the Klotho KL-VS allele, which increases secretion of Klotho *in vitro*, obtained better results in various cognitive tests, including verbal capacity, executive function, visual-spatial processing, and learning [6]. A meta-analysis of the KL-VS variant indicated that it is associated with healthy aging [13]. Finally, recent studies also suggest that Klotho might prevent the development of Alzheimer's disease (AD) associated with aging, probably by inhibiting insulin/IGF-1 signaling and, consequently, oxidative injury in the brain in a murine model of AD [14–16]. In agreement with this, the concentration of the extracellular Klotho domain is significantly diminished in the cerebrospinal fluid (CSF) of AD patients [17].

Unfortunately, little is known about s-KL, the secreted αKlotho. To address this, we have generated a new antibody (Ab-K113) against the exclusive peptide at the C-terminus of s-KL, and we demonstrate that the *klotho* transcript produced by alternative splicing generates a stable protein, which can be directly detected in protein extracts. We have also analyzed *klotho* expression in the CNS during pathological (AD) and non-pathological aging processes, as well as during aging in animals exposed to continuous moderate exercise, and we have identified a strong correlation between high expression levels of the two *klotho* transcripts and the healthy status of the animals during aging.

## Material and Methods

### Animals

The 3xTg-AD mouse strain harboring the familial AD mutations PS1/M146V, AßPPSwe, and tauP301L were genetically engineered at the University of California Irvine [18]. Male and female 3xTg-AD mice from the Spanish colony of homozygous 3xTg-AD mice, established in the Medical Psychology Unit, Autonomous University of Barcelona, were used in the present study. The Non-Transgenic mouse colony had the same hybrid genetic background (129 X C57BL/6) as 3xTg-AD. Genotypes were confirmed by PCR analysis of DNA obtained from tail biopsies. Animals were maintained in Macrolon cages under standard laboratory conditions of ad libitum food and water, 22±2°C, and 12-h light:dark cycles. Animals were under deep anesthesia before euthanased by decapitation. Experiments and necropsies were performed in the Central Animal Facilities of the Universitat Autonoma de Barcelona. This study was carried out in strict accordance with the current Spanish national regulations. The protocol was approved by the Committee on the Ethics of Animal Experiments of the Universitat Autonoma de Barcelona (CEEA-UAB) (Permit Number: 2196).

### Animal experimentation and sample collection

Four to five animals from different litters, grouped by genotype and gender, were housed in a cage with one running wheel (Activity Wheel Cage System for mice, Techniplast, Buguggiate, Italy). The system allowed continuous recording of the wheel turn number and therefore calculation of the average mouse running activity per cage. We chose to house the animals in groups to avoid the distress of individual housing. Periodic visual inspection suggested that all animals used the wheel, but it was not possible to quantify the running time of each mouse. Sedentary control mice were housed in similar cages without a running wheel. At specific times, animals were euthanized and samples from different brain areas (prefrontal cortex, cerebral cortex, hippocampus, and cerebellum) and the kidney were collected and transferred to tubes, snap frozen in liquid nitrogen, and stored at -80°C until RNA or protein extraction.

### Analysis of mRNA levels by quantitative qPCR

Total RNA was obtained from brain/kidney samples and extracted from the tissue samples using the QIAzol Lysis Reagent (Qiagen, USA), then quantified on a NanoDrop spectrophotometer (Thermo Scientific, USA.), and reverse transcribed into cDNA with iScript cDNA Synthesis Kit (Bio-Rad, USA). Gene-specific primers used for the qPCR analysis were: s-KL Fwd: 5'-TGGCTTTCCTCCTTTACCTG-3'; s-KL Rv: 5'-GCCGACACTGGGTTTTGT-3'; m-KL Fwd: 5'-TTCAAACCCGGAAGTCTTTG-3'; m-KL Rv: 5'-CCAGGCAGACGTTCACATTA-3'; m36B4 Fwd: 5'-ATGGGTACAAGCGCGTCCTG-3'; m36B4 Rv: 5'-AGCCGCAAATGCAGATGGATC-3'. Primers were initially optimized by standard PCR using Taq DNA polymerase (Qiagen) and were visualized by ethidium bromide staining on a 2% low melting agarose gel. Quantitative qPCR was performed on a Bio-Rad CFX-384 PCR machine at the Analysis and Photodocumentation Service of the Universitat Autonoma Barcelona using iTaqTM Universal SYBR Green Supermix (Bio-Rad). A two-step PCR reaction was carried out as follows: One cycle of 98°C for 2 minutes followed by 40 cycles of 95°C for 5 seconds and 58°C for 30 seconds. Samples were run as duplicates. The analysis of qPCR output data followed the manufacturer-suggested ΔCt method. Cycle thresholds (Ct) were measured, and the relative expression of genes was calculated by comparison of Ct values. All samples were normalized to m36B4, which was used as a reference gene. Melt-curve analysis was used to confirm the production of a single amplicon for each gene tested. Based on the RT-qPCR assay efficiency, gene amplification at a level higher than 35 cycles (ΔCt of 15) was considered to have no expression. A “no template control” was also included in each run.

### Anti-Klotho antibody characterization

The Ab K113 rabbit polyclonal antibody was produced by EZBiolab (Carmel, USA) using the designed immunogenic peptide VSPLTKPSVGLLLPH as the antigen. Samples were homogenized in lysis buffer (50 mM Tris-HCl pH 7.4, 150 mM NaCl, 1 mM ethylenediamine tetraacetic acid [EDTA], 1% NP-40, 0.25% sodium deoxycholate and Protease Inhibitor Cocktail Set I (Millipore, USA), sonicated, and finally centrifuged at 12,000g and 4°C for 15 minutes. Protein concentration was determined using the Pierce BCA Protein Assay (Thermo Scientific) using bovine serum albumin as the standard and a NanoDrop 1000 UV/Vis spectrophotometer (Thermo Scientific). Protein extracts (15–25 μg per sample) were run in denaturing acrylamide gels, and then electrotransferred to PDVF membranes (GE Healthcare, Spain). Membranes were blocked with TBS-T (20 mM Tris-HCl pH 7.5, 150 mM NaCl, 0.2% Tween-20) containing 5% skimmed milk, and incubated with the indicated primary antibody. Detection was performed with an appropriate horseradish peroxidase-conjugated secondary antibody (EZBiolab) and enhanced chemiluminiscence reagent (GE Healthcare). The K113 antibody was used at 1/5000 dilution; polyclonal rabbit anti-actin antibody (Sigma A2066, USA) at 1/1000; and secondary rabbit HRP-anti-Ig antibody (Dako-Cytomation, P0399, Denmark) at 1/10,000. Band pixel intensities were quantified using ImageJ (Wayne Rasband National Institutes of Health, USA) and normalized to actin levels.

### Cell culture and transfection

Human HEK-293 cells (QBiogene, USA) were cultured in six-well plates with Dulbecco’s modified Eagle’s medium (DMEM; Lonza, Switzerland) supplemented with 10% fetal bovine serum (FBS; Biowest, France) and antibiotics at 37°C and 5% CO_2_. Transfections were performed in 60–70% confluent cultures using 6 μg of the appropriate DNA complexed with polyethyleneimine (PEI 25 kDa; Aldrich, USA) per 10^6^ cells. Cells were harvested 48 hours after transfection, and mRNA and proteins were extracted.

### Statistical analysis

Data are presented as mean values ± SEM. Statistical calculations were performed using the G-Stat version 2.0 and Prism 5.04 statistical programs. Statistical significance between individual groups was determined by the Student's t test (unpaired, two-tailed) or one-way ANOVA followed by Tukey post-hoc analysis. In all the statistical analyses, p<0.05 was considered significant.

## Results

**Fig 1:**
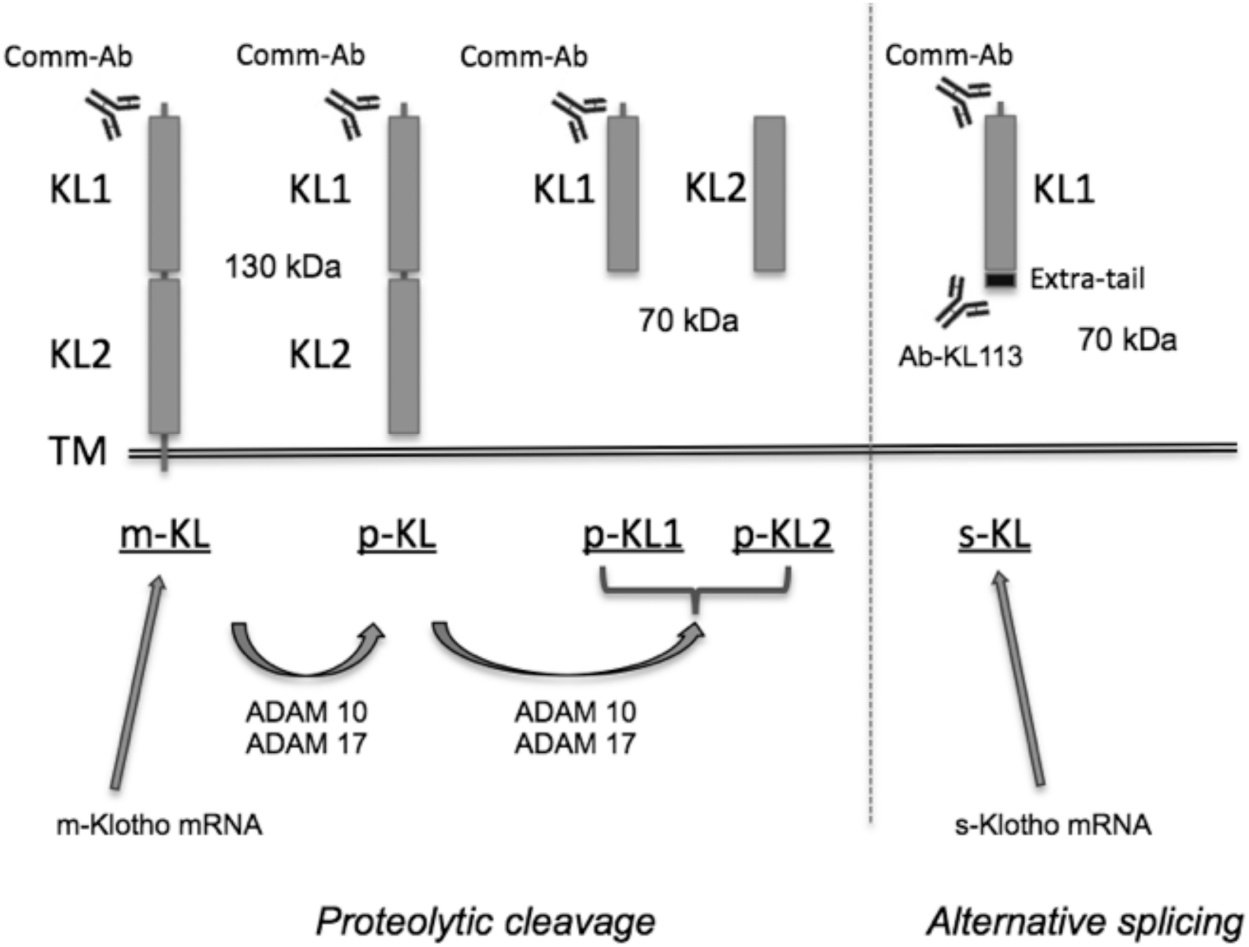
Scheme of the two Klotho isoforms. One of the transcripts encodes the transmembrane form (m-KL), which is processed by ADAM10 and ADAM17 α-secretases to produce proteolyzed Klotho (p-KL) that contains both KL1 and KL2 domains, or proteolyzed individual Klotho domains (p-KL1 and p-KL2). A second *klotho* mRNA transcript encodes the secreted form of Klotho (s-KL), containing only the KL1 domain plus an extra short C-terminal tail. Ab-KL113 specifically recognizes the exclusive extra C-terminal tail of s-KL. Commercial antibodies (Comm-Ab) are raised against the KL1 or KL2 domains (most frequently against KL1) and, therefore, they detect several Klotho forms. The different forms of the αKlotho protein are underlined.

The αklotho gene encodes two transcripts, one generating a 130-kDa transmembrane protein, and an alternative splice variant that generates a 70-kDa secreted protein. This alternative mRNA includes a specific secretion signal consisting of a 15 amino acid tail that is not found in the m-KL transcript [19, 20]. The extracellular domain of the transmembrane form can be cleaved by metalloproteinases ADAM10 and ADAM17 [21], which results in another form of soluble Klotho of about 130 kDa, which has been detected in serum, urine [22], and cerebrospinal fluid [4] (Fig 1). Moreover, two recent studies have indicated that there is a second recognition site for these proteases located between the KL1 and KL2 domains, which generates two new 70-kDa isoforms, one containing the KL1 domain only (similar to the s-KL isoform generated from alternative splicing, but without the specific amino acid tail), and the other one containing the KL2 domain [23, 24]. Thus, Klotho protein might enter the circulatory system through two main mechanisms: (a) from alternative splicing, and (b) by proteolytic cleavage mediated by ADAM10 and 17. However, the percentage at which each of these events occurs is unknown. This variety of Klotho isoforms has led to confusing nomenclature, and has made it difficult to associate physiological roles with the different Klotho isoforms. To address this, here we use the nomenclature proposed by Forster et al. [25]: m-KL stands for the full-length, transmembrane form; p-KL stands for proteolyzed Klotho, which is generated by cleavage at the short transmembrane domain; and s-KL stands for secreted Klotho, which is generated by alternative splicing.

### Secreted αKlotho and transmembrane αKlotho have a different spatial and temporal expression profile in the brain

**Fig 2:**
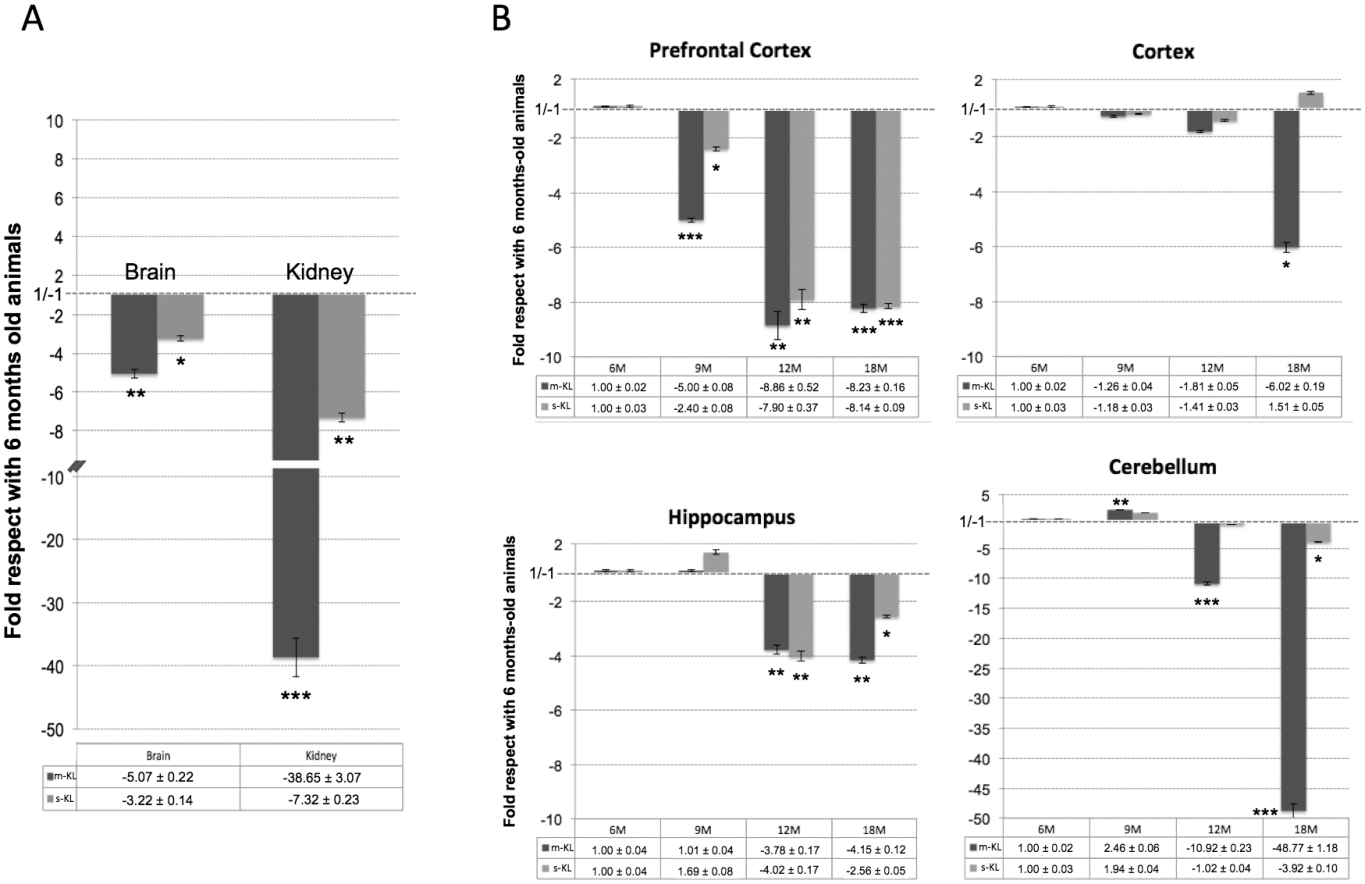
Analysis of the expression levels of the transmembrane Klotho (m-KL) and secreted Klotho (s-KL) transcripts by qPCR during aging. (**A**) Levels in the kidney and whole brain of 18-month-old mice compared with 6-month-old mice. (**B**) Spatial and temporal analysis of the m-KL and s-KL transcripts. The prefrontal cortex, cortex, hippocampus, and cerebellum of 6-, 9-, 12-, and 18-month-old animals were analyzed. m36B4 was used as a reference gene. N = 5 to 7; *p<0.05 **p<0.01 **p<0.001 by one-way ANOVA with Tukey post-hoc analysis). Error bars indicate SEM.

Most attention has been paid to the transmembrane form (m-KL), but not to the secreted form of Klotho (s-KL). In order to elucidate whether the expression profile of both Klotho proteins is similar, we first analyzed their expression in the kidney and brain because these are the two organs with highest expression [1]. Since we are interested in neurodegenerative diseases associated with aging, we then studied their expression profile in adulthood from young adult animals (6 months old) to elderly animals (18 months old). As observed in Fig 2A, expression levels of the two Klotho transcripts decay with age. This decay was especially pronounced in the kidney (38.6- and 7.3-fold for m-KL and s-KL, respectively), but is also statistically significant in the brain (5.1-fold for m-KL and 3.2-fold for s-KL). Moreover, as described previously [26], we found that in the kidney, the expression of m-KL was higher than that of s-KL (78-fold in 6-month-old mice, and 7-fold in 18-month-old mice; data not shown). Similarly, the expression of m-KL in the brain was also higher than of s-KL (9-fold in 6 months-old mice, and 5-fold in 18 month-old mice).

Due to the structural and functional heterogeneity of the brain, we analyzed whether the reduced expression of *klotho* in the brain associated with aging was uniform throughout the brain or was more pronounced in some areas than in others. We analyzed the expression in the prefrontal cortex, cortex, and hippocampus because of their role in learning and cognition, as well as in the cerebellum, because of the high expression of *klotho* described in Purkinje cells [8]. In all the areas analyzed, the expression of both *klotho* transcripts decayed with aging (Fig 2B). Notably, this decay was not uniform throughout the brain, and in addition, the decay of s-KL had a different profile to that of m-KL suggesting different regulation of their expression, and/or possible different roles for the Klotho proteins. In the prefrontal cortex, as early as 9 months, s-KL and m-KL levels were reduced significantly compared with the levels of 6-month-old animals (almost 90% reduction in 12- and 18-month-old animals, p<0.001). In contrast, in the hippocampus, the levels of s-KL and m-KL were not significantly reduced until 12 months of age (about 75% reduction, p<0.01), while in the cortex, a significant reduction was seen only in 18-month-old animals for m-KL (about 85% reduction, p<0.05). On the other hand, in the cerebellum, the expression of m-KL was slightly increased in 9-month-old animals, but decreased rapidly at 12 and 18 months of age (10-fold and 48-fold reduction, respectively, p<0.001), while the expression levels of s-KL were quite constant until the age of 18 months, when they were significantly lower than those in 6-month-old animals (4-fold reduction, p<0.05), but this decrease was much lower than that observed for m-KL.

### Generation of a specific antibody against the secreted αKlotho protein

**Fig 3:**
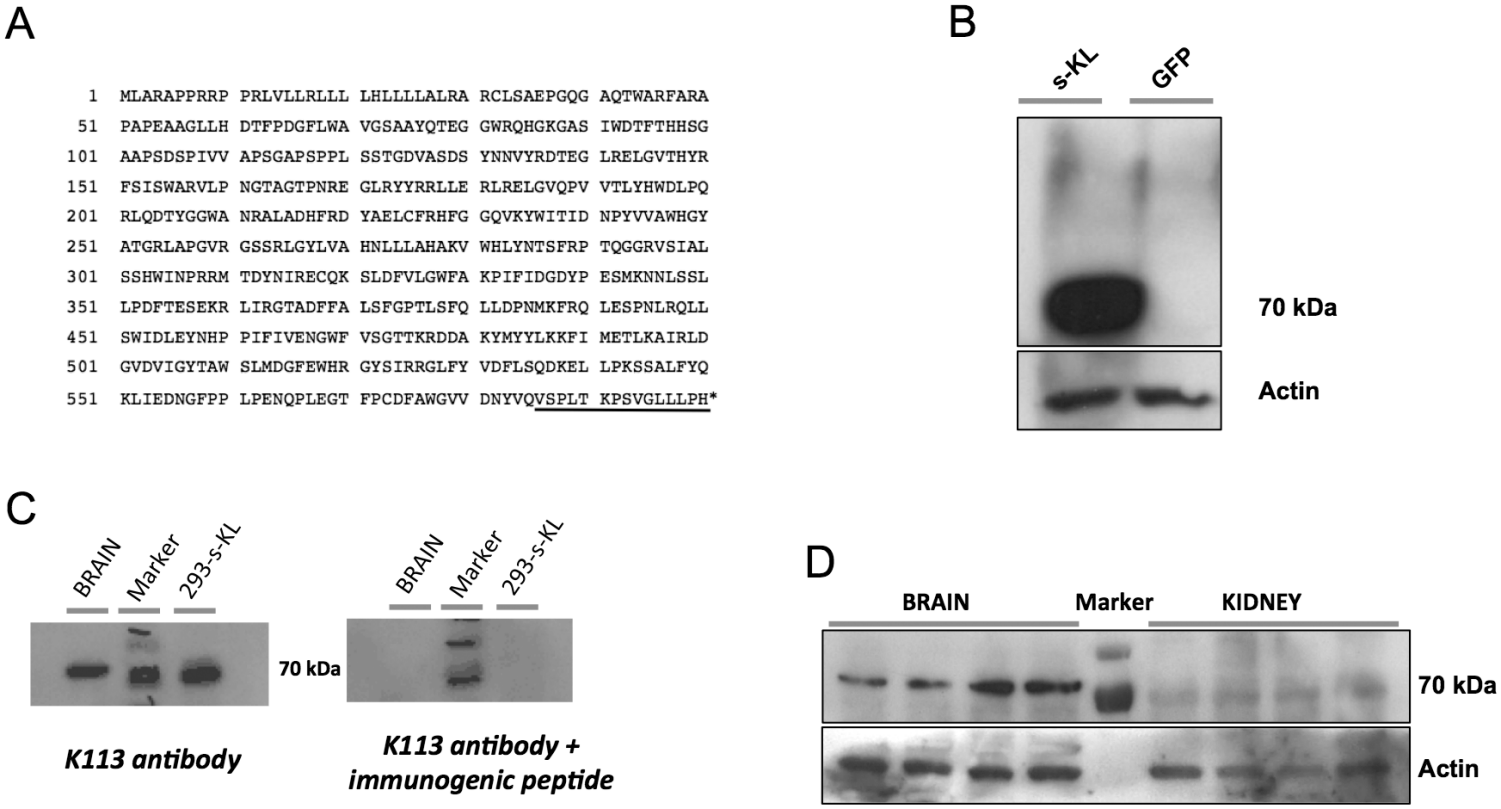
**(A)** Amino acid sequence of the s-KL transcript. The unique 15-aa tail is underlined **(B)** Immunoblot analysis of HEK293 cells overexpressing s-KL protein. As a control, lysates from HEK293 cells overexpressing GFP are shown. **(C).** Validation of the Ab-K113 antibody. Immunoblot analysis of lysates from murine whole brain and s-KL-transduced HEK293 cells overexpressing s-KL (293-s-KL). Right panel shows the corresponding immunoblot after incubating the membrane with the antibody and the immunogenic peptide. **(D).** Immunoblot analysis of the s-KL protein from brain and kidney of 6-month-old animals using the K113 antibody. 25 μg of protein extract was loaded per well. Actin levels are shown as the loading control.

Currently, the available anti-Klotho antibodies recognize epitopes that are common for both the secreted and the transmembrane Klotho proteins (including the soluble cleaved Klotho) (Fig 1). To specifically analyze the levels of the secreted Klotho protein, we generated an antibody against the exclusive peptide (15 amino acids for murine s-KL, and 16 amino acids for human s-KL) at the C-terminus of the s-KL protein (Fig 3A).

By using different immunogenicity sequence analyses (Chon & Fasman Beta-Turn Prediction, Kolaskar & Tongaonkar Prediction, Antigenicity Parker Hydrophilicity Prediction, and Emini Surface Accessibility Prediction), a high antigenicity was predicted for the SPLTKPSVGLLLPH epitope. We then blast-searched (http://blast.ncbi.nlm.nih.gov/Blast.cgi) for other proteins containing this epitope. Only the secreted Klotho protein had 100% identity. Finally, a specific antibody (Ab K113) against s-KL was generated in rabbit. As seen in Fig 3B, the K113 antibody detected a band of approximately 70 kDa in HEK293 cells transduced with the s-KL cDNA, but not in HEK293 cells transduced with the GFP gene. The expected size of s-KL is 62.3 kDa. The K113 antibody was further validated by competition with the immunogenic peptide in s-KL-transduced HEK293 cells, as well as in brain tissue (Fig 3C). Of note, in contrast to the transmembrane Klotho isoform, the secreted isoform was hardly detected in the kidney, but was mainly detected in the brain (9-fold higher in whole brain than in kidney; Fig 3D) suggesting its activity is basically performed in the brain.

### Detection of the secreted αKlotho protein in different brain areas of young and old adult mice

**Fig 4:**
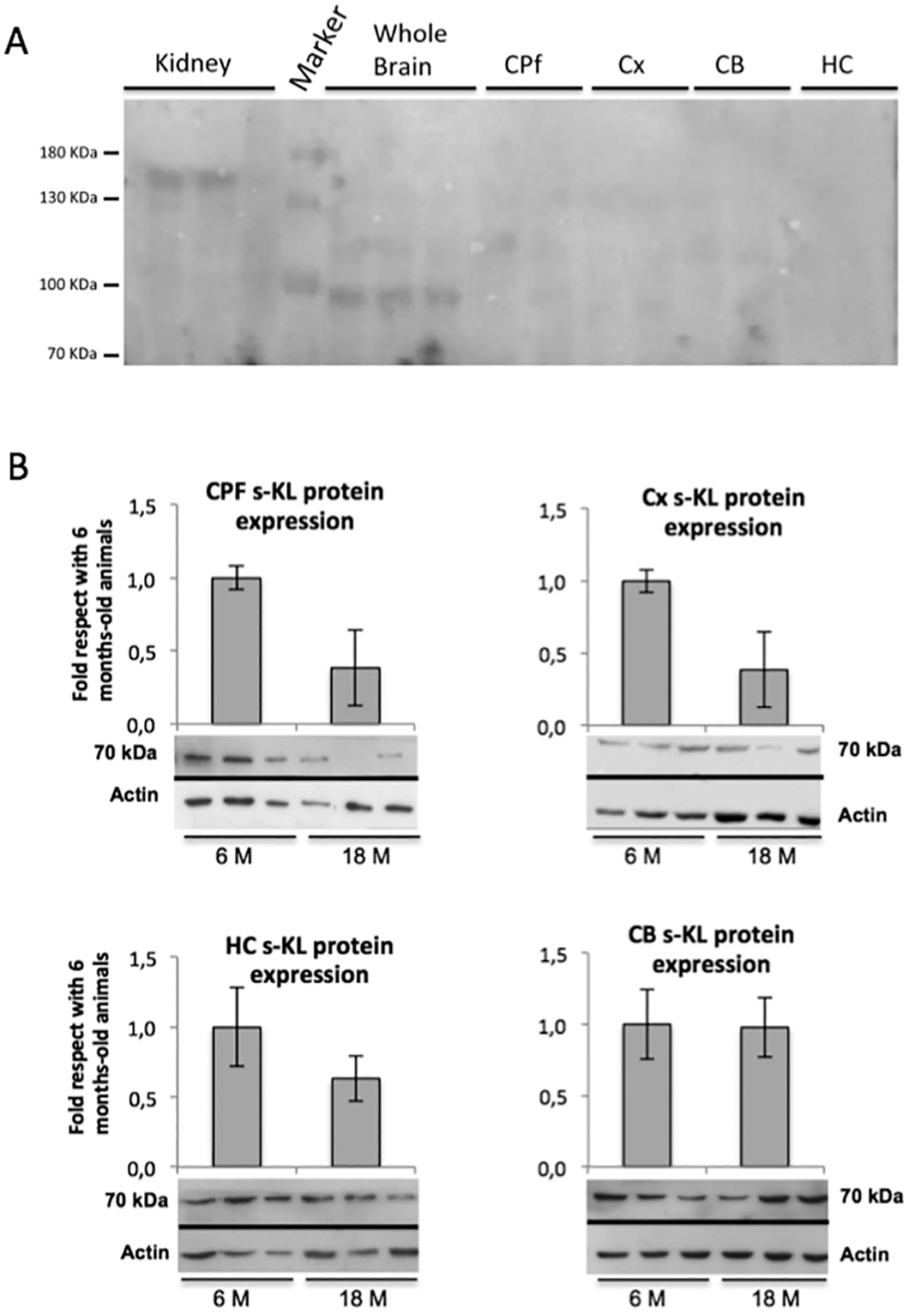
Analysis of the Klotho protein during aging by immunoblotting. **(A)** Immunoblotting of Klotho by antibody KM2076 in kidney, brain and brain areas. **(B)** The prefrontal cortex (CPF), cortex (CX), hippocampus (HC), and cerebellum (CB) of 6- and 18-month-old C57BL/6 mice were analyzed (n = 6 to 10). Immunoblots were quantified by densitometry, and columns show the corresponding values. Results from 18-month-old mice relative to those obtained from 6-month-old mice are shown. Actin was used to normalize the amount of protein analyzed.

The levels of the secreted Klotho protein were analyzed in the prefrontal cortex, cortex, hippocampus, and cerebellum of 6-month-old and 18-month-old mice using the K113 antibody. Unfortunately, in brain areas we could only evaluate s-KL but not m-KL, because despite the commercial KM2076 antibody detects Klotho in whole brain, it is not able to detect it in any of the brain areas analyzed (Fig 4A).

In agreement with the levels of the s-KL mRNA transcript, the levels of the s-KL protein declined with aging in the prefrontal cortex, cortex, and hippocampus (down to 41%, 49%, and 63%, respectively, compared with 6 months-old animals) (Fig 4B). However, in the cerebellum, the s-KL levels were similar at both ages. Indeed, the correlation between mRNA and protein levels in the brain areas is difficult since proteins produced in other brain areas may be present because of axonal projections from distant neurons. Of note, this is the first time that the levels of the alternatively spliced secreted Klotho protein have been specifically quantified without interference from the KL1 or KL1+KL2 domains produced after cleavage of the transmembrane Klotho protein.

### Expression of Klotho in different brain areas decays more rapidly in the 3xTg-AD transgenic model of Alzheimer disease than in healthy animals

**Fig 5:**
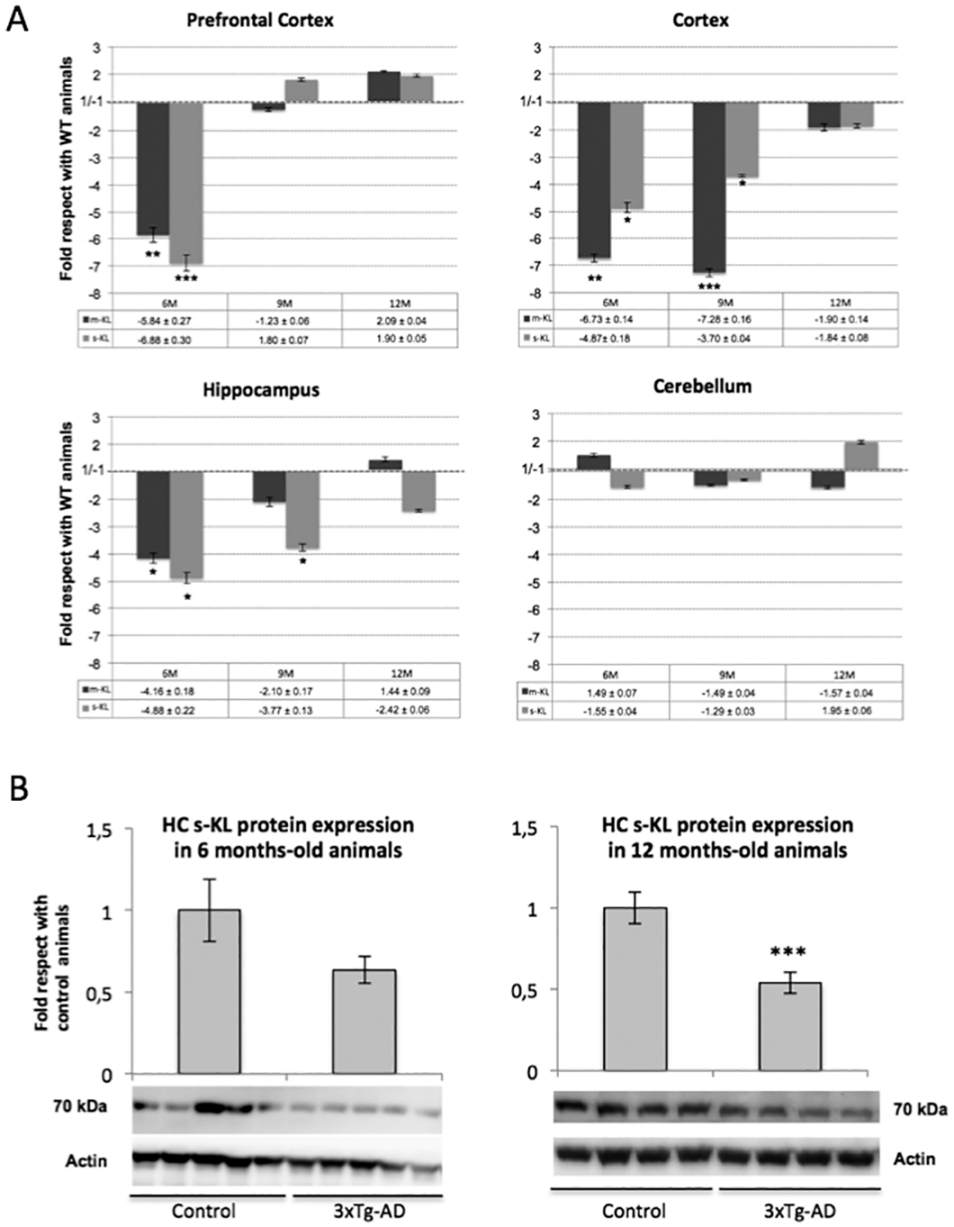
Analysis of the s-KL protein in 3xTg-AD mice. (A) qPCR analysis of the expression levels of the transmembrane Klotho (m-KL) and secreted Klotho (s-KL) transcripts in 3xTg-AD mice and control mice. s-KL and m-KL transcripts were quantified by qPCR from the prefrontal cortex, cortex, hippocampus, and cerebellum of 6-, 9-, and 12-month-old mice of the 3xTg AD or control mice. Results are compared with siblings not exposed to moderate exercise (WT). m36B4 was used as a reference gene. (B) Immunoblot analysis of s-KL from the hippocampus of 3xTg-AD mice using the K113 antibody. Actin was used to normalize the amount of protein analyzed. N = 6 to 10; *p<0.05 **p<0.01 **p<0.001 by one-way ANOVA with Tukey post-hoc analysis for qPCR, and Student's t-test for immunoblotting. Error bars indicate SEM.

We also analyzed whether the reduction observed in the expression of the s-KL and m-KL transcripts in the different brain areas during non-pathological aging was also observed in pathological aging as happens in neurodegenerative disorders like Alzheimer's disease. We analyzed the expression profile of the s-KL and m-KL transcripts in the prefrontal cortex, cortex, hippocampus, and cerebellum of 6-, 9-, and 12-month-old 3xTg-AD mice. As observed in non-pathological aging, the expression of both transcripts declined rapidly, but compared with control animals of the same age, in the 3xTg-AD mice this decline was exacerbated earlier, and was significantly different at 6 months of age in the prefrontal cortex, cortex, and hippocampus (Fig 5A). However, during aging, differences in the expression profile of s-KL and m-KL were reduced, and at 12 months of age, s-KL and m-KL levels were similar in 3xTg-AD and control mice. Interestingly, in the cerebellum, where the effects of the Alzheimer's disease are more discrete than in other areas, the expression of s-KL and m-KL declined at all ages at a similar rate than in control animals.

As observed for s-KL mRNA expression, levels of the s-KL isoform were also lower in 3xTg-AD mice. Thus, compared with aged-matched controls, s-KL protein levels in the hippocampus were reduced to 63% (p = 0.2) and 53% (p<0.001) at 6 and 12 months of age, respectively (Fig 5B), whilst in other areas, such as the cerebellum, s-KL levels were reduced to 31% at 12 months of age (p<0.05%), but were not reduced at 6 months of age (data not shown).

### Voluntary moderate continuous exercise in adulthood prevents the decline in expression of the s-KL and m-KL transcripts

**Fig 6:**
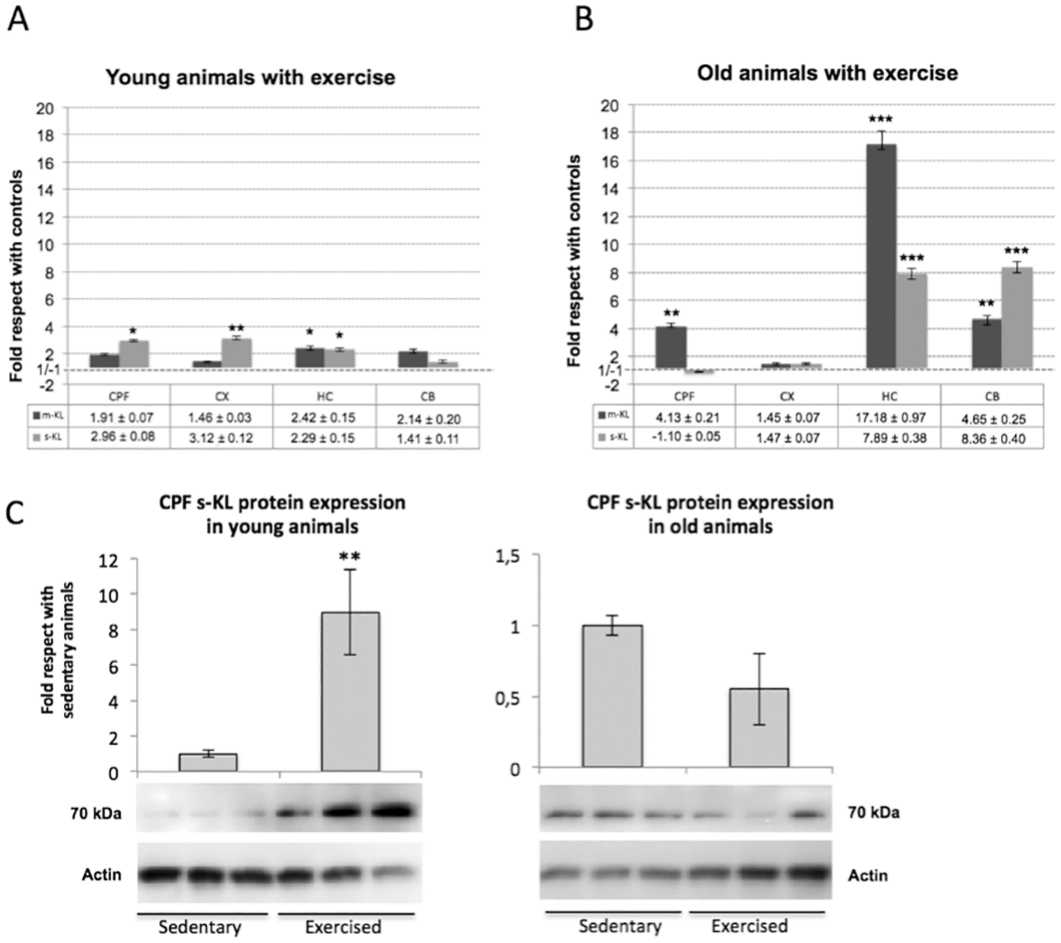
qPCR analysis of the expression levels of m-KL and s-KL transcripts in animals exposed to continuous moderate exercise. **(A)** Young C57BL/6 mice started daily exercise at 6 months of age, and **(B)** old C57BL/6 mice started daily exercise at 12 months of age. Moderate exercise was performed daily throughout the experiments (6 months) using a running wheel. Animals of the young group were sacrificed at 12 months of age, and animals of the old group were sacrificed at 18 months of age. s-KL and m-KL transcripts from the prefrontal cortex, cortex, hippocampus, and cerebellum were quantified by qPCR. Results are compared with siblings not exposed to moderate exercise. m36B4 was used as a reference gene. (C) Immunoblot analysis of s-KL from the prefrontal cortex and hippocampus of sedentary and exercised mice using the K113 antibody. Actin was used to normalize the amount of protein analyzed. N = 6 to 8; *p<0.05 **p<0.01 **p<0.001 by one-way ANOVA with Tukey post-hoc analysis for qPCR, and Student's t-test for immunoblotting. Error bars indicate SEM.

Moderate continuous exercise is considered healthy for the organism and, especially in adulthood, may help to prevent, among others, muscular and cardiovascular problems. In fact, some authors have hypothesized that the Klotho protein may respond or may be affected by physical activity, since Klotho effects are mediated via growth factors such as IGF-1 [27]. Moreover, a recent report also suggested an important link between Klotho deficiency and age-associated muscle deterioration [28], while aerobic exercise increases plasma Klotho levels [29]. We analyzed whether continuous exposure to a healthy lifestyle during aging could affect or maintain the expression levels of the s-KL and m-KL transcripts in the CNS. Two groups of mice of 6 and 12 months of age were exposed daily to the running wheel for 6 months (from 6 to 12 months of age), and their expression profiles were compared to control mice of the same age that did not exercise. In animals that underwent moderate continuous exercise, s-KL and m-KL expression levels were higher than in control animals (Fig 6A and 6B), suggesting a positive correlation between health status and higher expression levels of s-KL and m-KL. Of note, although this correlation was observed in young and old animals, this effect was more pronounced in old mice (12 months of age), reaching, for example, levels of 8- and 17-fold higher expression of s-KL and m-KL, respectively, in the hippocampus of exercised mice compared to not exercised or sedentary controls. However, the s-KL and m-KL expression profiles were not uniform throughout the different brain areas analyzed, which indicates not only temporal but also differential spatial regulation in the brain of the two *klotho* transcripts. Interestingly, despite the old mice being exercised less than 50% that of young mice (data not shown), the effect, except in the cortex, was more significant in older mice. Surprisingly, the levels of the s-KL protein were not significantly increased by continuous exercise either in young or old animals, except in the prefrontal cortex, where in young animals, the s-KL levels were increased by 9-fold (p<0.01; Fig 6C). Future experiments analyzing differences in the mechanisms regulating the activity of the s-KL isoform (mRNA expression, mRNA stability, protein stability, etc.), or in the regional distribution of the secreted Klotho isoform in the CNS should be performed to identify the main causes explaining the discrepancy observed between s-KL mRNA and s-KL protein levels.

## Discussion

To date, several studies have analyzed the expression profile of *klotho* in different organs. However, despite Klotho being first described as an anti-aging factor, most of the reported studies have been done in fetal, postnatal, or young animals. Moreover, although brain expression of *klotho* has been suggested to be important to CNS aging [1], only Duce et al. have studied *klotho* expression in the whole brain throughout adulthood (from 6- to 24-month-old mice; B6D2F1 strain), and they reported a reduction of 2- to 3-fold in the *klotho* mRNA during aging when analyzing whole brain hemispheres [30], which is in the same range observed by us during aging in several structures of the brain of C57BL/6 mice. Similar results (about a 2-fold reduction in *klotho* mRNA) were also described in the white matter of aged rhesus monkeys [30].

Concerning studies of *klotho* expression at the spatial level, despite Klotho protein being detected by immunoblotting in total brain homogenates [31], evidence of Klotho in specific brain areas is still limited [32]. This may be the result of deficient detection tools, lower overall *klotho* expression, and/or poor transcription of *klotho* mRNA in the brain [5]. However, in order to understand the function of s-KL in the brain, it is interesting to study how *klotho* expression patterns vary across brain regions. In this regard, in rats, m-KL levels are fairly stable in the developing hippocampus, whilst in the cortex, m-KL mRNA levels are high during the first week of life, drop from P10 to P21 and then markedly increase by adulthood, suggesting that Klotho plays distinct roles at different points in neural development, as well as within different brain regions during these times [5].

With the aim of determining whether the transcription of the two *klotho* transcripts (m-KL and s-KL) is similar or is differentially regulated during non-pathological aging and pathological neurodegenerative aging, we evaluated their expression in the CNS in aged, AD, and exercised mice. As previously reported [20], we also found that the levels of the m-KL transcript were higher than those of s-KL in the murine brain and kidney. In addition, when we analyzed their transcription levels in different brain areas, we observed that the expression profile of *klotho* was not uniform throughout the brain, and moreover, the expression levels declined significantly during aging in all areas analyzed. On the other hand, despite both transcripts having similar spatio-temporal expression profiles, they were not identical, which may suggest different roles. During non-pathological aging, in the cerebellum, m-KL levels are reduced by 10-fold and 50-fold in mice of 12 and 18 months of age, respectively, while s-KL levels were not reduced at 12 months of age, and were reduced by only 3-fold at 18 months of age. Since the cerebellum and kidney are the sites where *klotho* is highly expressed, the difference between m-KL and s-KL expression levels might indicate different physiological functions for s-KL and p-KL. To this end, additional studies (addressing other issues, such as differences in subcellular distribution or in protein stability) should also be performed to elucidate whether s-KL and p-KL have different functions or if the two isoforms are functionally identical.

In this regard, K113 antibody may be a valuable tool, not only for Western blots, but also for development of an ELISA. The K113 antibody can facilitate the analysis of p-KL and s-KL functions since their current quantification using antibodies or ELISA against the common KL1 domain cannot be considered accurate as performed thus far, and therefore, further studies need to be done in order to know whether some of the functions currently associated with the proteolyzed p-KL are, in fact, associated with the secreted s-KL. More importantly, the K113 antibody has also shown that although the mRNA levels of s-KL in the brain and kidney are similar, the s-KL protein is about 10 times more abundant in the brain than in the kidney. This unexpected difference suggests that while kidney s-KL might be rapidly secreted into the bloodstream, the brain s-KL seems to accumulate within the CNS parenchyma, although other possibilities cannot be discarded, such as renal s-KL also accumulating in the brain, or the presence of factors that stabilize Klotho in the brain.

*Klotho* is an amyloid precursor protein (APP)- and APP-like Protein 2 (APLP2)-dependent gene that is regulated by the large secreted ectodomain fragment soluble (APPsβ) [33], and therefore, it may play a role in the development of Alzheimer’s disease. In fact, recent studies have shown that Klotho might prevent the development of Alzheimer's disease associated with aging by inhibiting insulin/IGF-1 signaling and, consequently, oxidative injury in the brain [14–16]. This might explain why chronic administration of ligustilide prevents (via *klotho* upregulation) the development of AD-like neuropathologies and memory impairment [14]. Indeed, inhibition of the insulin-like signaling pathway is also a mechanism for antiaging and lifespan extension. However, the mechanism by which Klotho inhibits activity of insulin/IGF-1 receptors in the brain remains to be determined [34].

Thus far, several studies have analyzed how alterations in Klotho levels affect insulin, IGF1, mTOR-mediated signaling, and Wnt-mediated signaling, as well as oxidative stress, nitric oxide production, activity of ion channels, oligodendrocyte differentiation, development of Alzheimer's disease, and neuroprotection processes in brain. However, little is known about how different genetic and/or external environmental factors affect Klotho functions. We have studied whether *klotho* expression in the CNS was influenced during aging by a pathological process, such as Alzheimer's disease, and whether a healthy lifestyle, such as voluntary moderate continuous exercise, could also influence *klotho* expression. Interestingly, we observed a strong correlation between high expression levels of the two *klotho* transcripts (m-KL and s-KL) and the health status of the animals during aging. In the 3xTg-AD mouse model of Alzheimer's disease, both, the mRNA and protein levels of *klotho* declined more rapidly than in age-matched control animals. Since this decline was more evident in middle-aged animals than in old animals, and was not uniform in the different brain areas analyzed (prefrontal cortex, cortex, hippocampus, and cerebellum), this may help to discern what proportions of the decline in *klotho* expression are associated with aging and with Alzheimer's disease. Equally important, moderate continuous exercise performed either in young or middle-aged adults resulted in a long-term reduction in the decline of *klotho* expression in the CNS, both in terms of level and speed of progression, although it did not stop or revert the decline. However, at the protein level, these effects were significant only in the prefrontal cortex and not in the other brain areas analyzed. Interestingly, just 3 months of such moderate continuous exercise was enough to improve the performance in learning and memory tasks in both the 3xTg-AD mice and controls [35], with oxidative stress as one of the central targets for such neuroprotection [36]. In the present work, it is not possible to conclude whether higher expression levels of *klotho* in the CNS are a cause or a consequence of the healthier status of the animals. In fact, probably both options are valid, since Klotho seems to work through a synergistic loop between (a) high levels of Klotho expression inducing beneficial effects, and (b) a healthy lifestyle increasing the levels of Klotho expression in the CNS.

Taking into account that the brain is one of the organs where *klotho* is highly expressed and its neuroprotective role—together with the fact that the secreted isoform, but not the transmembrane isoform is the main Klotho isoform expressed in humans [20]—the evidence of a specific brain regional and temporal distribution of s-KL protein seems to be of functional relevance. In this regard, we believe that at least s-KL (and likely also p-KL), may have an active role in neuronal activity, and especially in learning and memory processes, in these key areas for cognitive function, which are targets of study both in aging and neurodegenerative processes. Similarly, it will be relevant to investigate the expression profile of s-KL in other neurodegenerative diseases, as well as to specifically knock-down or knock-in its expression in models of pathological and non-pathological aging to better describe the functions of s-KL not only in specific areas of the brain, but also in other organs.
